# Influence of Fabric Weave on Thermal Radiation Resistance and Water Vapor Permeability

**DOI:** 10.3390/polym12030525

**Published:** 2020-03-01

**Authors:** Ana Kiš, Snježana Brnada, Stana Kovačević

**Affiliations:** 1 Textile Company Čateks d.d., 40000 Čakovec, Croatia; a.kis@cateks.hr; 2 Department of Textile Design and Management, Faculty of Textile Technology, University of Zagreb, Prilaz baruna Filipovića 28a, 10000 Zagreb, Croatia; stana.kovacevic@ttf.hr

**Keywords:** aramid fibers, fabric weave structures, radiant heat protection, porosity, water vapor permeability

## Abstract

In this work, aramid fibers were used to develop new, high-performance fabrics for high-temperature protective clothing. The research was based on the impact of the weave structure on fabric resistance to radiant heat. The goals of the research were primarily related to the development of new fabric structures created by the weave structure, which gives better protection of the body against high temperatures in relation to the standard weave structures that are used today. According to the results obtained it can be concluded that the fabric weave significantly affects the fabric structure, which consequently determines the effectiveness of protection against high temperatures. The justification for the use of multi-weft and strucks weave structure, which provides greater thermal protection and satisfactory breathability than commonly used weave structures, was ascertained.

## 1. Introduction

The protection of the human body from extreme external conditions has been known since ancient times. The manufacture of fabrics resulted in their widespread use in households, as well as the usage of clothing for protection against extreme temperatures, precipitation, wind, UV radiation, mechanical shocks, etc. The selection of raw materials in the manufacturing process of protective clothing has to contribute to the most efficient protection. With technological development, as well as the invention of man-made fibers, the development of protective fabrics has changed significantly, affecting their manufacturing for targeted applications [1,2,3]. The requirements placed on textile materials for high-temperature protective clothing have led to innovations in the development and production of woven fabrics [4,5,6].

Numerous requirements and high standards are placed on protective textile materials. Meeting those demanding requirements leads to protection of the human body in the ultimate and extreme conditions. By using new raw materials, different fabric structures, and surface treatments, adequate body protection from extreme conditions can be achieved. Other than thermal protection, protective material must provide comfort while at the same time providing impeccable mobility of the body when worn. The fabrics used for high-temperature protective clothing are most commonly woven in simple weave structures such as plain, twill and satin weaves, which are then bonded to the composite with other sheet materials, e.g., knitted fabrics, semi-permeable membranes, etc. [7,8,9,10,11].

Several parameters of textile materials directly or indirectly affect the amount of thermal protection. These can be categorized as the chemical and morphological identity of the material, physical properties of the material, structural properties and finishing. Each of these parameters depends on the type of fiber, the characteristics of the yarn, i.e., the spinning technique, the construction and structure of the fabric, and its finishing process. The development of effective fire protecting textile material begins with the appropriate selection of the raw material, i.e., textile fiber, which must have high performance in fire retardancy and resistance. The minimum amount of oxygen required for the combustion of a fiber is different for different fiber types [12], and this value is defined by the limiting oxygen index (LOI). Fire-retardant/-resistant fibers are those with a LOI value greater than 21%. Through chemical modifications of first-generation natural fibers such as wool, cotton, and viscose, and second-generation synthetic fibers like polyester, polyamide and acrylic fiber, the LOI values can be improved. These types of fibers are referred to as chemically modified fire-retardant fibers [13,14,15]. A special group of synthetic fibers are inherently fire-resistant fibers that are not easily flammable or meltable. [13,16]. They are structurally arranged in such a way that they can inherently resist fire. Some of the most commonly used inherently fire-resistant polymeric fibers are p-aramid fiber, m-aramid fiber, polyimide fiber, PBI fiber, polyacrylate, glass fiber, ceramic fiber, etc. These fibers are produced by modifying synthetic polymers at the molecular level, which provides a thermally stable fiber structure. Using these fibers, further finishing processes of textile material are not necessary, and it retains fire-resistant properties even after the maintenance processes. Using fire-resistant and fire-retardant fibers, the development of fabrics for thermal protective clothing is possible [17]. Composites from a combination of multiple components are most commonly used to produce thermal protective clothing [18]. There are three major components that can be used as a part of these composites: shell fabrics, thermal liners, and moisture barriers. The shell fabric is usually a woven fabric from aramid fiber, PBI fiber, or blends [19]. Thermal liners are mostly made by combining the nonwoven and woven fabrics, both made from fire-retardant/resistant fibers, and joined into the laminate. Moisture barrier fabric is manufactured by coating the woven or nonwoven base with a chemical substance such as neoprene, polyvinyl chloride, polyurethane or polytetrafluoroethylene, or by laminating the woven/nonwoven base with semi-permeable membrane [20,21,22].

In [23], the authors found that air efficiency in thermal protection was provided by the use of textile composite materials of which the front layer was a woven fabric in plain, twill, or basket weave. A large amount of air and its position in the interstices of the composite materials, as well as the presence of less moisture, increases the thermal protection. According to [24], raw material composition, structure, thickness and mass of the fabric affect thermal protection. The greater thickness or fabric mass provides better thermal protection. The impact of the weave structure was not extensively investigated, and the results did not reveal clear influences. The authors focused more on the effect of surface coating, while weave structures were less investigated. The authors of [25] studied the thermal protection of fabrics that are commercially used for the manufacture of protective clothing for firefighting as single-layer and multilayer fabrics. The research focused on the effect of fabric mass and thickness on thermal resistance, air permeability, evaporation resistance and water spreading rate. Some studies have dealt with the categorization of commercial fabrics woven in different weave structures [26].

Woven fabrics that are used at the face of the composite material for high-temperature protection are individually porous and breathable. However, a textile composite that contains a layer of a semi-permeable membrane and layers that are bonded by a hot melt adhesive loses its breathability properties. This can result in reduced comfort and even cause some hazards like the thermal stress of the carrier. Therefore, the aim of this research is to develop woven fabrics of complex structures in which the presence of air pockets and increased thickness provide satisfactory thermal protection while ensuring good breathability. Their protective properties will be based primarily on the use of different types of weave structures, which are nowadays not commonly used for protective fabrics. Weave structures that can provide higher effectiveness in protection against high temperatures will be selected. The use of the strucks weave, which is known for its “crimps” on the surface of the fabric, and for its specific threads that, by their function and properties, additionally crimp the fabric, create a partially thick and breathable structure. Because air is a good heat insulator, the efficiency of the protecting strucks weave fabrics against high temperatures was investigated. By using weave structures that increase fabric thickness, such as multi-weft woven fabrics, one compact fabric was created. It is expected to provide good thermal protection and porosity, which also means breathability. The manufacture of multi-weft woven fabrics allows an unlimited design of fabric structures, but at the same time, it presents a great challenge for their development and production. Finally, the effectiveness of the protection of newly developed fabrics was investigated and compared with the requirements for the materials used today. The above-mentioned considerations point to the possibility of achieving progress in the functionality and justification of the use of innovative fabrics for thermal protective clothing, which has so far not been explored in the review of the available literature.

## 2. Materials and Methods

### 2.1. Materials

Investigations of different types of fabrics woven (produced at the Faculty of Textile Technology University of Zagreb, on the Fully-automatic rapier sample loom, Fanyuan Instrument (HF) co., Ltd., Hefei, China) from the same warp and weft yarns were carried out: yarn fineness is 16.7 × 2 tex, composition: 95% M-aramid Conex NEO + 5% P-aramid Twaron MOK3. The number of twists per meter was 323.2 with a correlation coefficient of 4.4% and S twist direction. The yarn breaking force was F = 617.1 cN, CV = 5.8%, elongation at break ε = 20.2%, CV = 11.3%. The warp density did not change in the reed. The weave structure and warp tension, which was kept constant during the weaving process affected the weft density. The basic parameters of the fabric samples are presented in Table 1.

Warp and weft density and fabric thickness were measured 24 h after the removal of the fabric from the sample weaving machine equipped with CAD/CAM weaving system. Eight heald frames were used for all weave structures, heald frame sett was 15 dents/cm, denting was carried out by one warp thread being drawn into one dent.

The weave structures and the corresponding cross-sections are shown in Table 2. According to the selected weave structures and cross-sections, the structure of fabrics, which differ in thickness and surface crimps, can be observed, resulting from different interlacing of warp and weft threads. In multi-weft weave fabrics, the weft prevails on the fabric face and back, which means that the transverse system of threads is more important for the final protective properties. In plain and twill weave fabrics the significance of warp and weft threads is equal in their protective roles. The strucks weave on the fabric face is created by the plain weave, three free weft threads are in crimps, and floating warp threads prevail on the fabric face. This is the reason a special fabric structure is created where individual warp threads occasionally float and occasionally interlace in plain weave. The consequence is a difference in the tension between threads during the weaving process resulting in crimps on the fabric surface.

### 2.2. Methods

- Fabric volume porosity

Woven fabric porosity refers to the void fraction or total void space within the volume of the woven material. The volume porosity of a woven fabric *P* can be theoretically calculated based on volume fulfillment which expresses the percentage of thread volume within the fabric volume.

Fabric porosity was calculated according to Equations (1) and (2) [27,28,29].

(1) $$P=\left(1-\frac{q_{fabr.}}{q_{fibr.}}\right)\cdot 100\%.$$

where *q_fabr_*_._ stands for volume density of the fabric and *q_fibr_*_._ is the specific fiber density which for meta aramid fiber equals 1.38 g/m^3^.

Respectively,

(2) $$q_{fabr.}=\frac{M\;\left(\frac{g}{m^{2}}\right)}{T\;\left(mm\right)\cdot 1000}$$

where *M* is for a surface mass of fabric in g/m^2^, and *T* is fabric thickness in mm.

- The resistance of fabrics to radiant heat

The main function of firefighters’ clothing is to resist heat transfer from the thermal environment to the wearer body to protect it from burn injuries. Radiation heat transfer was tested according to EN ISO 6942 2003, method B, with a heat flux of *Q*_0_ = 20 kW/m^2^. The testing was performed on a device manufactured according to the regulations in the standard. The let-through heat flux density across the sample using a calorimeter was calculated according to Equation (3).

(3) $$Q_{c}=\frac{M_{bp}\cdot Cp\cdot 12}{A\cdot \left(t_{24}-t_{12}\right)}=\frac{66,131}{\left(t_{24}-t_{12}\right)}$$

where *M_bp_* is the mass of copper plate and equals 0.036 kg, *C_p_* is the specific heat of copper which is 0.385 kJ/kg °C, *A* is area of the copper plate and equals 0.002515 m^2^ and *t*_12_, *t*_24_ are the time required for a temperature rise of 12 °C and 24 °C, respectively.

Heat transfer factor expresses the heat flow passing during 1 h through 1 m^2^ of fabric with actual thickness, and temperature difference of two media 1 °C. High *TFQ*_0_ indicates a good heat transfer (low thermal stress, i.e., good low-energy comfort at ordinary working conditions), while low heat transfer factor indicates a good insulation. The factor of heat transfer was calculated according to Equation (4):

(4) $$TFQ_{0}=\frac{Q_{C}}{Q_{0}}$$

where *Q_C_* is leaked heat flux density through the sample to calorimeter in kW/m^2^ and *Q*_0_ is default heat density (radiation source per calorimeter) in kW/m^2^.

- Water vapor permeability and resistance to the passage through the material

Today, it is increasingly demanding, more complex and difficult to achieve certain properties of fabrics or composites, which are prescribed by standards for a particular purpose, and it is even more difficult to achieve the sustainability of these properties during use. High-temperature fabrics with high levels of protection must have comfort properties. By testing water vapor permeability and resistance to the passage through materials, their breathability or wear comfort can be defined.

Measurement of water vapor permeability of textiles is done according to standard ISO 15496.

Water vapor permeability (*WVP*) of the sample is calculated according to Equations (5)–(7):

Δ*m* = *m*_15_ − *m*_0_ (5)

(6) $$WVP_{app}=\frac{\Delta m_{app}}{a\cdot \Delta_{p}\cdot \Delta_{t}}$$

Water vapor permeability through the material can be calculated according to Equation (7):

(7) $$WVP=\left(\frac{a\cdot \Delta_{p}\cdot \Delta_{t}}{\Delta_{m}}-\frac{1}{WVP_{app}}\right)-1$$

Water vapor is transported from a region with high water vapor pressure to a region with lower water vapor pressure. The high water vapor pressure is maintained by a water surface kept at a constant temperature. The water vapor pressure (*p_sb_*) is calculated by the following Formula (8), showing that the pressure (*p_sb_*) is highly dependent on the water bath temperature (*Tb*):

(8) $$\begin{array}{cc} p_{sb}=133.3 & \times (\frac{2919.611}{\left(Tb+273\right)}-\;4.79518\times \log\left(Tb+273\right)\\ & +23.03733) \end{array}$$

The relative humidity (*RH*, %) of a water surface can be considered to be 100%.

The lower water vapor pressure is maintained in the air inside the pores of the membrane by a saturated aqueous solution of potassium acetate kept at a constant temperature. The relative humidity *RH* on temperature *Ta* is calculated by the following Formula (9):

(9) $$RH=22.4388+0.156288\cdot Ta-\left(0.612868\cdot 10^{-2}\right)\cdot Ta^{2}$$

The water vapor pressure (*p_sa_*) is calculated according to Formula (10):

(10) $$\begin{array}{cc} p_{sa}=133.3 & \times (\frac{2919.611}{\left(Ta+273\right)}-4.79518\times \log\left(Ta+273\right)\\ & +23.03733) \end{array}$$

The partial water vapor pressure difference (∆*p*) is calculated by the following Formula (11):

(11) $$\Delta p=p_{sb}-\frac{p_{sa}\cdot RH}{100}\;\left(Pa\right)$$

At the lower temperature, the pressure gradient is also lower, giving a lower transport of water vapor, which implies a smaller mass differential to be weighed (∆*m*), thus decreasing the accuracy of the test method. This is the reason in International Standard ISO 15496 the water bath temperature was chosen as 23.0 °C, and the tolerance allowed is only ± 0.1 °C.

From the obtained values of water vapor permeability fallowing by Formula (5), the water vapor resistance (*R_et_*) can be determined according to Formula (12). The greater the amount of water vapor transported through the sample, the lower the resistance to water vapor transmission will be. The connection between WVP and Ret represents the latent thermal surface (*Lt*) of water evaporation at the test temperature water bath 23 °C.

Determination of water vapor resistance (*R_et_*) is calculated according to Equation (12):

(12) $$R_{et}=\frac{1}{WVP\cdot L_{t}}$$

Latent heat of water evaporation at a temperature of 23 °C:

*L_(t)_* = (2500.8 − (2.36∙T) + (0.0016∙T^2^) − (0.00006∙T^3^))
*L_(23°C)_* = 2446.64 J/g
1 J = Ws
*L_(23°C)_* = 2446.64 Ws/g
*L_(23°C)_* = 0.67962 Wh/g

## 3. Results and Discussion

According to the test results of fabric resistance to radiant heat, a significant difference among weave structures can be found (Table 3).

The fabric in a 4-weft weave provided the highest resistance to radiant heat, the let-through heat flux averaged 6.10 kW/m^2^ and the heat transfer factor was 0.3 kW/m^2^. It took 12.37 s for a calorimeter temperature rise of 12 °C, while it took 23.23 s for a calorimeter temperature rise of 24 °C. The second highest resistance to radiant heat was with the fabric in a 2-weft weave. Although its thickness was more than twice as small, its mass and density were only 25% less than in case of the 4-weft weave fabric, and resistance to radiant heat was expectedly good, amounting to *Qc* = 7.63 kW/m^2^. From Figure 3 it can be observed that by increasing the surface mass of the sample, the resistance to thermal protection increases, i.e., in this case, the value of *Qc* decreases.

The strucks weave fabric ranked third by its resistance to radiant heat. The let-through heat flux density amounted to *Qc* = 8.87 kW/m^2^, and the heat transfer factor amounted to *TFQ*_0_ = 0.43. Despite its surface unevenness and visible pores among fabric crimps, it provided good protection against radiant heat. By further development of this weave structure, it will be possible to avoid prominent pores and thus further improve its radiation protection against heat.

The satin and twill weaves are ranked right after the strucks weave. They had similar resistance to radiant heat: *Qc* = 8.85 kW/m^2^ for the satin weave, and *Qc* = 9.67 kW/m^2^ for the twill weave. Heat transfer factor for the satin weave was *TFQ*_0_ = 0.44, and for the twill weave *TFQ*_0_ = 0.48.

Plain weave was the weakest weave structure regarding the effectiveness of resistance to radiant heat. With its compact structure, the plain weave achieved *Qc* = 11.56 kW/m^2^, and the heat transfer factor was *TFQ*_0_ = 0.58.

Test results of water vapor permeability and resistance to the passage through materials are shown in Table 4 and the parameters were calculated according to Equations (5) to (12).

Water vapor permeability ranged from *WVP* = 0.3935 g/m^2^Pah for 4-weft weave to *WVP* = 1.5481 g/m^2^Pah for strucks weave. The resistance to the passage of water vapor is inversely proportional to water vapor permeability and ranged from *R_et_* 0.95 g/m^2^W for strucks weave to *R_et_* 3.74 g/m^2^W for 4-weft weave. The water vapor permeability results are correlative with the results of the sample mass differences before and after the testing. The larger the mass difference, the greater the water vapor permeability, and ranged from Δ*m* = 1.1981 g for the 4-weft weave to Δ*m* = 3.3645 g for strucks weave.

Table 5 shows the summary results of sample testing.

The highest porosity was found in 4-weft weave (0.8880), followed by the strucks (0.8852), which has the highest values of thickness and mass. The fabrics woven in 4-weft and sturcks weave structures contain a higher amount of air, so-called “pockets”, which will create an additional shield as a heat insulator. The thinnest and most light weighted fabrics are those in plain and twill weaves, with the lowest porosity, an which are nowadays mostly used for high-temperature protective clothing, often as an integral part of multilayer composite material. Four-weft weave fabrics, as well as strucks weave fabrics, have a greater mass which increases their thickness and the amount of air in the fabric structure as essential parameters in protection against high-temperatures. The strucks weave structure is ahead of other weave structures because its crimps considerably increase the thickness and porosity with only a small increase in the mass.

**Figure 1:**
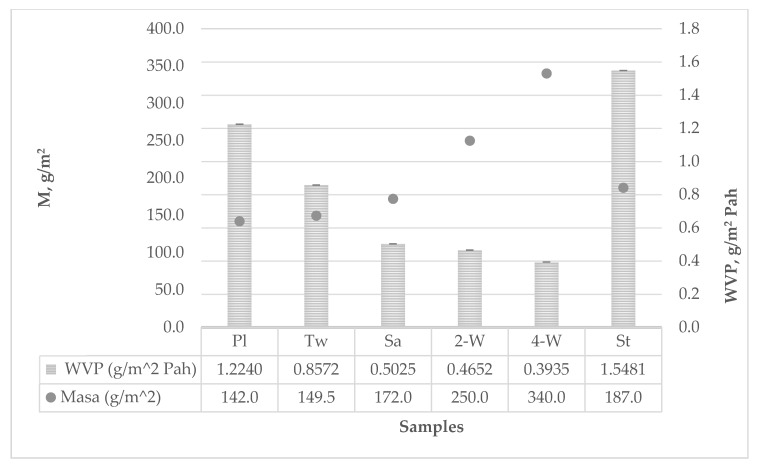
Water vapor permeability diagram.

The graph in Figure 1 shows that water vapor permeability is reduced by increasing the surface mass of the sample, with the exception of the fabric in the strucks weave where the water vapor permeability is the highest of all samples, although the surface mass is slightly higher than two-dimensional fabrics (plain, twill and satin weave). This is due to the specific fabric structure of the more voluminous domain for enhanced macro-scale crimp of the fabric, while the interlacing segments in the plain weave make it more porous and more breathable. Regardless of the differences, all fabric samples have satisfactory breathability compared to composite materials intended for firefighter protective clothing. Considering that plain and twill weave fabrics are often used as a part of composite laminated materials, by increasing the number of layers and in the presence of hot melt adhesive, the water vapor permeability will also increase significantly, *R_et_*.

**Figure 2:**
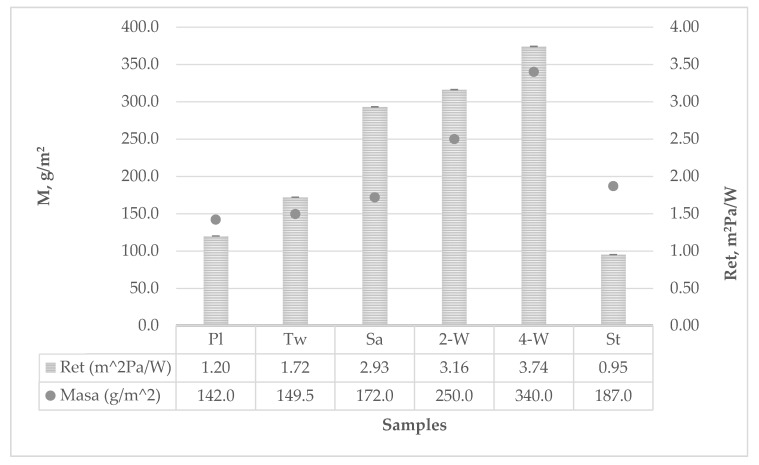
Water vapor resistance diagram.

The graph in Figure 2 shows the water vapor resistance of the fabric samples. A high, positive, linear relationship between the mass of the fabric and the water vapor resistance can be observed. The exception is the strucks sample and the reason for that is its specific structure.

**Figure 3:**
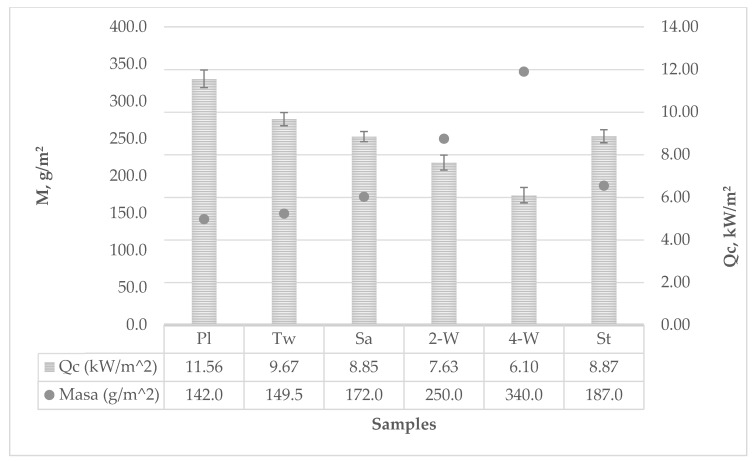
Leaked heat flux density through the sample to the calorimeter diagram.

The graph in Figure 3 shows that the leaked heat flux density through the sample to the calorimeter is correlated with surface mass only in two-dimensional samples, while in the more complex structures there is a weaker relationship between these two parameters, and for more accurate prediction the additional parameter of the structure should be introduced.

**Figure 4:**
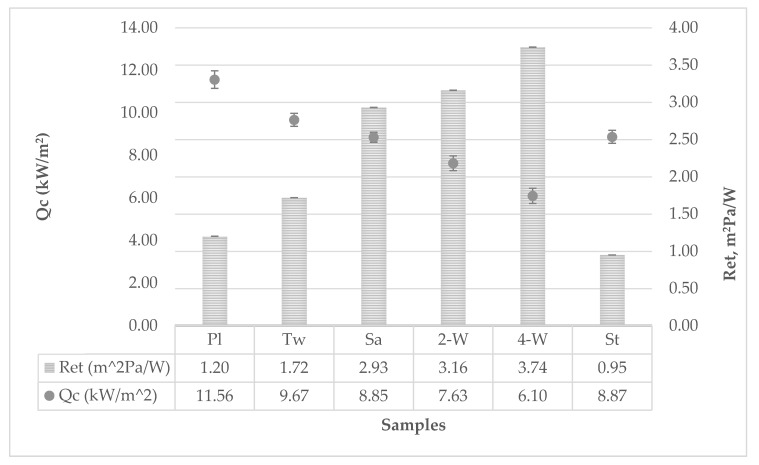
Vapor resistance–Leaked heat flux density through the sample to the calorimeter diagram.

The graph in Figure 4 shows a relatively good correlation between the parameters of the water vapor resistance diagram and leaked heat flux density over the sample to the calorimeter. Increasing values of water vapor resistance decrease the leaked heat flux density through the sample.

Table 6 shows the correlation factors between the tested fabric parameters.

The mass of the fabric is a parameter that shows very good correlation with the most important parameters related to heat protection (*M/Qc* = −0.92 and *M/TFQ*_0_ = −0.91) and the parameters of ‘breathability’ (*M/WVP* = −0.58 and *M/R_et_* = 0.78). Given that the mass is derived from the fabric structure itself, this is further evidence that the fabric construction and weave itself affect the properties of the fabrics addressed in this paper. Furthermore, the correlation factors show a moderate correlation between the porosity of the fabric with *Porosity/Qc* = −0.52 and *Porosity/TF* = −0.55, while there is no correlation with the permeability and water vapor resistance (*Porosity/WVP* = 0.04; *Porosity/R_et_* = 0.15).

**Figure 5:**
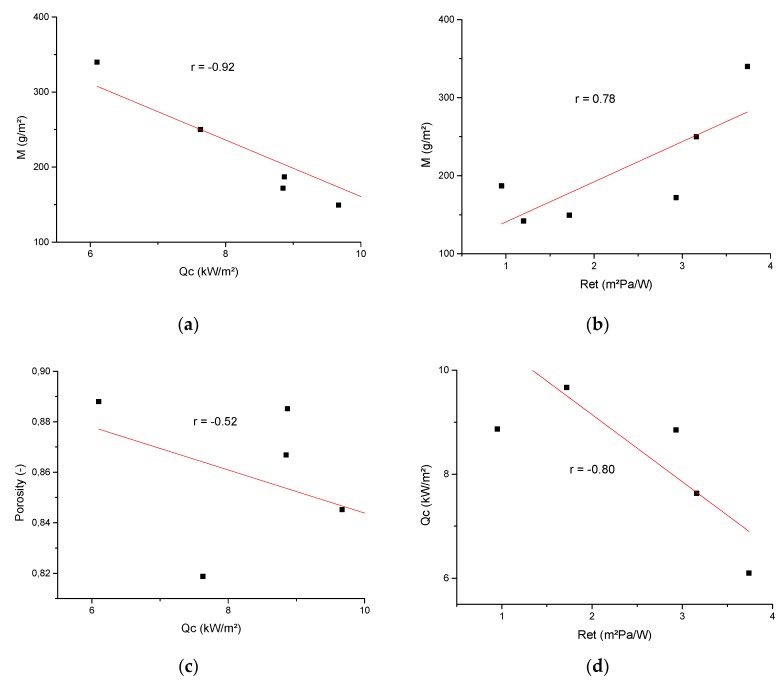
Plots with highly correlated parameters followed by linear fit and regression analysis: (**a**) Mass—Leaked heat flux density over sample to calorimeter; (**b**) Mass—Water vapor resistance; (**c**) Fabric volume porosity—Leaked heat flux density over sample to calorimeter; (**d**) Leaked heat flux density over sample to calorimeter—Water vapor resistance.

Figure 5 shows the scatter plots and the linear relationship of the parameters that are most closely related (Table 6). Graphic representations are accompanied by (Table 7) parameters of Pearson’s correlation coefficient, denoted by Pearson’s r, which is the measure of the strength of the linear relationship between paired data.

The graphs (Figure 5) and the table (Table 7) shows that the parameter of fabric mass correlates very well with the parameters of leaked heat flux density through the sample and water vapor resistance, and the linear function describes their relationship very well. Correlation between the parameters of leaked heat flux density through the sample and water vapor resistance is also high. The correlation of the fabric volume porosity and leaked heat flux density through the sample is moderate and more parameters need to be introduced to better describe their relationship.

**Figure 6:**
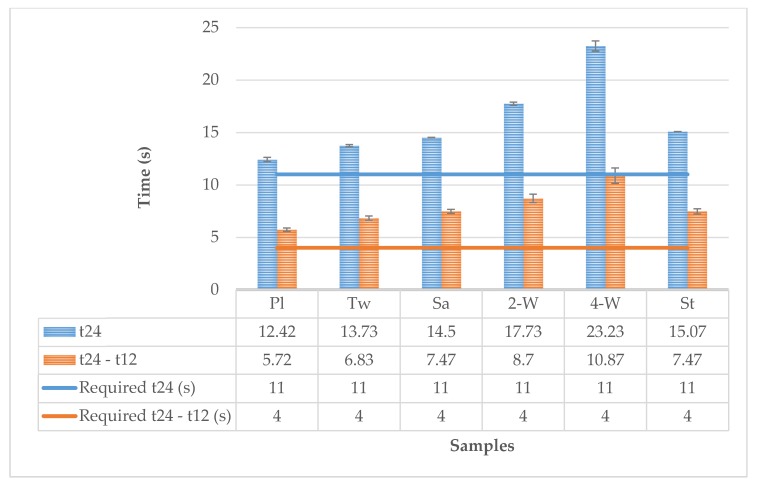
Required parameters for increasing the sample temperature to 24 °C and difference of measured times for a temperature rise of 24 °C and 12 °C compared to requirements according to the standard the EN 15614 Protective clothing for firefighters requirements for wildland clothing test methods and requirements.

Comparing the tested properties of the samples with the requirements described in the standard EN 15614 (Figure 6), it can be concluded that all the samples satisfy the requirement for heat transfer (radiation) through material, when tested following the Method B of EN ISO 6942:2002 with a heat flux density of 20 kW/m^2^ the single layer, the component assembly or multilayer clothing assembly shall have the minimum level as follows: RHTI 24 ≥ 11 s RHTI 24 − RHTI 12 ≥ 4 s.

**Figure 7:**
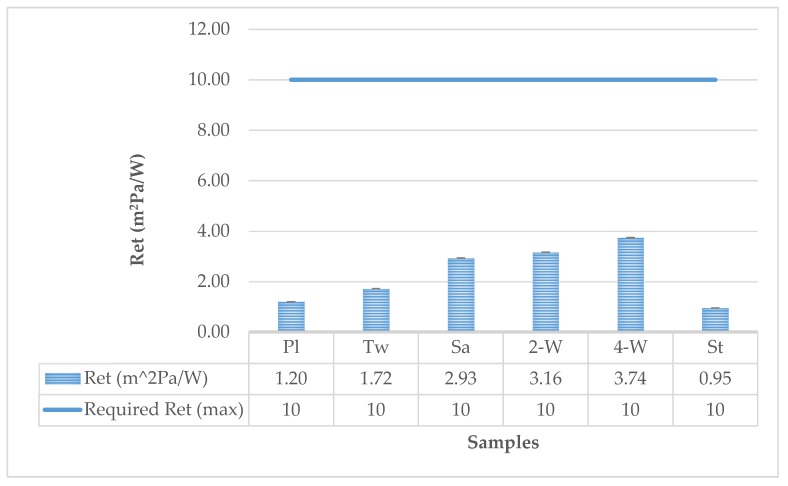
Vapor resistance compared to requirements according to the standard EN 15614 Protective clothing for firefighters requirements for wildland clothing test methods and requirements.

Comparing the tested properties of the samples with the requirements of the standard EN 15614 (Figure 7), it can be concluded that all the samples satisfy the requirement for water vapor resistance, where the mean water vapor resistance of material or material combination shall give a water vapor resistance ≤ 10 m^2^ Pa/W.

However, it can be observed that the complex woven structures, especially the 4-weft fabric, provide higher thermal protection with optimal breathability of the material, which is much higher than the one at the composite materials used for firefighters’ clothes. In this regard, the prototype of the fabric, with improved breathability than composite materials and a higher level of protection than single-layer woven materials, is developed.

## 4. Conclusions

Different types of weaves affect the fabric structural properties such as fabric thickness, surface mass, porosity, protection against radiant heat and water vapor permeability.

The structure complexity of woven fabric such as in the case of 4-pick and strucks weave fabrics results in an increase of their porosity, i.e., the amount of air in the fabric structure, as parameters of crucial importance for protection against high temperatures. The strucks weave structure, with its crimps, leads to a noticeable increase of fabric thickness with a slight rise of mass, which is an indicator of high porosity.

In the case of fabric resistance to radiant heat, a considerable difference can be found among the weave structures. The multi-weft weave fabrics (4-weft and 2-weft weave) and the strucks weave fabric provided the highest resistance to radiant heat. The two-dimensional weave structures: plain, satin and twill weave, provided less resistance to radiant heat. The 4-weft weave fabrics had the lowest water vapor permeability, whereas the strucks weave fabric had the highest water vapor permeability. Consequently, the strucks weave fabric proved to provide more breathability and wear comfort. However, fabric thickness had an influence on lower vapor permeability and greater resistance to water vapor passage through the fabric.

The best performance in terms of thermal protection and breathability was given by samples with complex woven structures, especially 4-weft sample followed by strucks weave sample. Further development of those structures and their proper choice for protection against high temperatures and high water vapor permeability will be continued.

## Figures and Tables

**Table 1:** Weave and density of fabrics samples.

Designation	Weave	Dwa (Threads/cm)	Dwe (Threads/cm)
2-W	2-weft weave	17	60
4-W	4-weft weave	17	80
Tw	Twill 2/2	17	24
St	Strucks weave	17	40
Pl	Plain weave	17	23
Sa	Satin weave	17	36

**Table 2:** Structures and cross-sections of fabric samples; weave name, weave unit, thread cross-section.

Weave Name	Weave Unit	Fabric Cross-Section
Plain weave	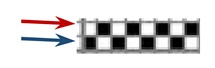	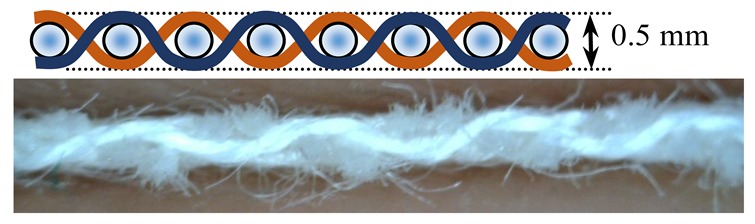
Twill 2/2	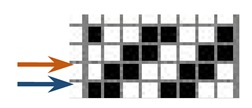	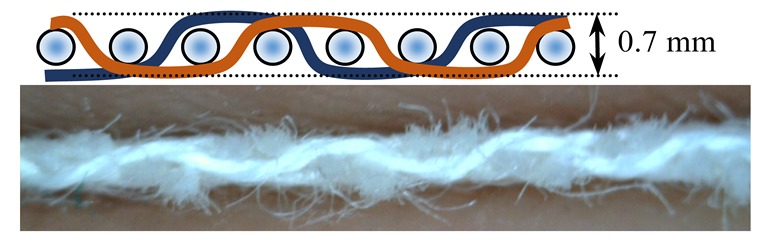
Satin weave	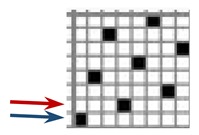	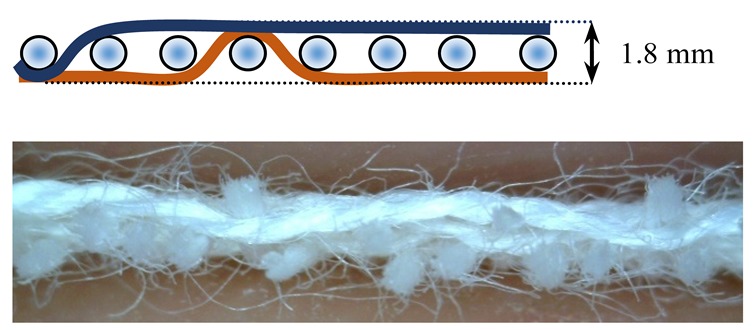
2-weft weave	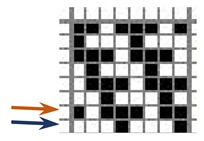	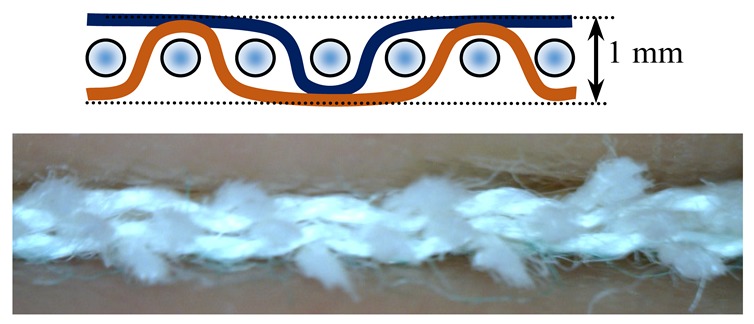
4-weft weave	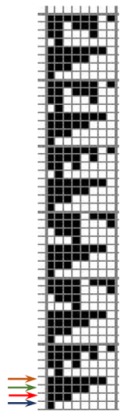	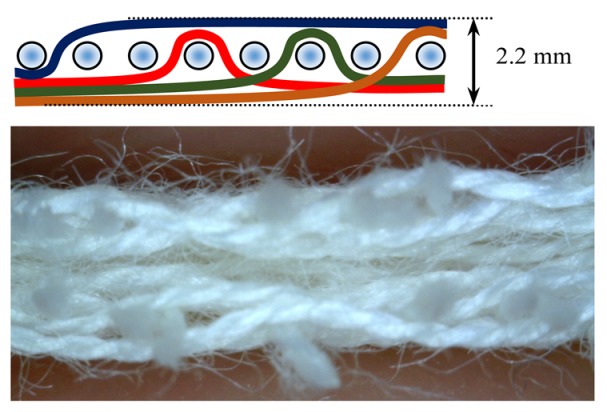
Strucks weave	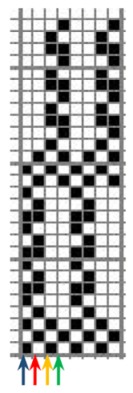	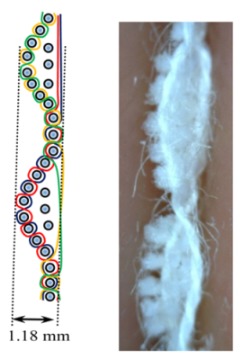

**Table 3:** Results of fabric resistance to radiant heat.

Designation	*t*_12_ (s)	*t*_24_ (s)	*t*_24_ − *t*_12_ (s)	*Qc* (kW/m^2^)	*TFQ*_0_ (-)
Pl	6.70	12.42	5.72	11,56	0.58
Tw	6.90	13.73	6.83	9.67	0.48
Sa	7.03	14.50	7.47	8.85	0.44
2-W	9.03	17.73	8.70	7.63	0.38
4-W	12.37	23.23	10.87	6.10	0.30
St	7.60	15.07	7.47	8.87	0.43

**Table 4:** Results of fabric samples water vapor permeability and resistance.

Designation	Δ*m* (g/m^2^Pah)	*WVP* (g/m^2^Pah)	*R_et_* (m^2^Pa/W)
Pl	2.8926	1.2240	1.20
Tw	2.2482	0.8572	1.72
Sa	1.4744	0.5025	2.93
2-W	1.3822	0.4652	3.16
4-W	1.1983	0.3935	3.74
St	3.3645	1.5481	0.95

**Table 5:** Results of fabric samples, summary table.

Designation	*Mass* (g/m^2^)	*T* (mm)	*Porosity* (-)	*WVP* (g/m^2^Pah)	*R_et_* (m^2^Pa/W)	*Qc* (kW/m^2^)	*TFQ*_0_ (-)
Pl	142.0	0.58	0.8214	1.2240	1.20	11.56	0.58
Tw	149.5	0.70	0.8452	0.8572	1.72	9.67	0.48
Sa	172.0	0.94	0.8668	0.5025	2.93	8.85	0.44
2-W	250.0	1.00	0.8188	0.4652	3.16	7.63	0.38
4-W	340.0	2.20	0.8880	0.3935	3.74	6.10	0.30
St	187.0	1.18	0.8852	1.5481	0.95	8.87	0.43

**Table 6:** Coefficients between pairs of data.

Property	*Mass* (g/m^2^)	*T* (mm)	*Porosity* (-)	*WVP* (g/m^2^Pah)	*R_et_* (m^2^Pa/W)	*Qc* (kW/m^2^)	*TFQ*_0_ (-)
*Mass* (g/m^2^)	1.00						
*T* (mm)	0.92	1.00					
*Porosity* (-)	0.39	0.70	1.00				
*WVP* (g/m^2^Pah)	−0.58	−0.40	0.04	1.00			
*R_et_* (m^2^Pa/W)	0.78	0.63	0.15	−0.95	1.00		
*Qc* (kW/m^2^)	−0.92	−0.87	−0.52	0.63	−0.80	1.00	
*TFQ*_0_ (-)	−0.91	−0.87	−0.55	0.60	−0.77	1.00	1.00

**Table 7:** Parameters of the strength of the linear relationship between highly correlated properties.

Parameter	*M* − *Qc*	*M* − *R_et_*	*Pv* − *Qc*	*Qc* − *R_et_*
Equation	y = a + bx			
Pearson’s r	−0.92	0.78	−0.52	−0.80

## Abbreviations

*A*	Area of the copper plate (0.002515)	m^2^
*a*	Area of the measuring cup opening	m^2^
*C* *_p_*	The specific heat of copper (0.385)	kJ/kg°C
*T*	Thickness of fabric	mm
*D_wa_*	Density of the warp threads	threads/cm
*D_we_*	Density of the weft threads	threads/cm
*L* *_t_*	Latent heat of evaporation of water at temperature t	Wh/g
*M*	Surface mass of fabric	g/m^2^
*M_bp_*	Mass of copper plate (0.036)	kg
*M*_0_	Mass of the measuring cup before testing	g
*M*_15_	Mass of the measuring cup after testing	g
*P*	Fabric volume porosity	(%)
*p_sa_*	Saturated water vapor pressure at the test room temperature T_a_	Pa
*p_sb_*	Saturated water vapor pressure at the water bath temperature T_b_	Pa
*R* *_et_*	Water vapor resistance	m^2^Pa/W
*RH*	Relative humidity in equilibrium with saturated potassium acetate solution	%
*RHTI*	Mean time measured	s
*RHTI*24 − *RHTI*12	A difference of measured times for temperature rise of 24 and 12 °C	s
*T* *a*	The temperature in the test room	°C
*T* *b*	The temperature in the water bath	°C
*T*_12_	The time required for the temperature rise of 12 °C	s
*t*_24_	The time required for the temperature rise of 24 °C	s
*TFQ*_0_	Heat transfer factor	kW/m^2^
*WVP*	Water vapor permeability of the specimen	g/m^2^Pah
*WVP* *_app_*	Apparatus water vapor permeability	g/m^2^Pah
*Q_C_*	Leaked heat flux density through the sample to the calorimeter	kW/m^2^
*Q*_0_	Default heat density (radiation source per calorimeter)	kW/m^2^
*q_fabr._*	Volume density of the fabric	g/m^3^
*q_fibr._*	Specific fiber density (M-aramid = 1.38 g/m^3^)	g/m^3^
Δ*M*	Change in the mass of the measuring cup during the period ∆t	g
Δ*m_app_*	Change in the mass of the measuring cup on the specimen holder with membrane only during the period of Δt	g
Δ*p*	Partial water vapor pressure difference across the specimen	Pa
Δ*t*	Measuring time	h
